# GAN inversion method of an initial in situ stress field based on the lateral stress coefficient

**DOI:** 10.1038/s41598-021-01307-1

**Published:** 2021-11-08

**Authors:** Li Qian, Tianzhi Yao, Zuguo Mo, Jianhai Zhang, Yonghong Li, Ru Zhang, Nuwen Xu, Zhiguo Li

**Affiliations:** 1 State Key Laboratory of Hydraulics and Mountain River Engineering, College of Water Resources and Hydropower, Sichuan University, Chengdu, 610065 China; 2 Power China Chengdu Engineering Corporation Limited, Chengdu, 610072 China

**Keywords:** Civil engineering, Solid Earth sciences

## Abstract

The initial in situ stress field influences underground engineering design and construction. Since the limited measured data, it is necessary to obtain an optimized stress field. Although the present stress field can be obtained by valley evolution simulation, the accuracy of the ancient stress field has a remarkable influence. This paper proposed a method using the generative adversarial network (GAN) to obtain optimized lateral stress coefficients of the ancient stress field. A numerical model with flat ancient terrain surfaces is established. Utilizing the nonlinear relationship between measured stress components and present burial depth, lateral stress coefficients of ancient times are estimated to obtain the approximate ancient stress field. Uniform designed numerical tests are carried out to simulate the valley evolution by excavation. Coordinates, present burial depth, present lateral stress coefficients and ancient regression factors of lateral stress coefficients are input to GAN as real samples for training, and optimized ancient regression factors can be predicted. The present stress field is obtained by excavating strata layers. Numerical results show the magnitude and distribution law of the present stress field match well with measured points, thus the proposed method for the stress field inversion is effective.

## Introduction

As a foundation for underground engineering design and stability analysis^1^, the initial in situ stress field of a rock mass refers to the natural stress state of the rock mass before engineering construction^2^, which has a great effect on the deformation and failure of the rock mass. To ensure the construction safety of engineering, it is necessary to obtain an optimized initial in situ stress field for deep-buried underground engineering^3^. The formation of an in situ stress field is a complex process that is affected by topography, geology, rock behavior, tectonic evolution and other factors^4–6^. Therefore, it is nearly impossible to obtain an optimized in situ stress field for every space point in underground engineering, but it is still important to obtain a more optimized initial stress field by various inversion methods.

In situ stress can be obtained directly by field in situ stress measurement methods, such as strain relief, acoustic emission, borehole cores, and hydraulic fracturing tests^7–11^. However, due to limited funding and testing technology, as well as complex geological tectonic and steep terrain conditions, it is hard to obtain a large number of in situ stress measurement results^12,13^. Moreover, the measured stress can only reveal the distribution characteristics of the stress field in a local area. Therefore, it is necessary to use an efficient back analysis method combined with advanced numerical simulation to derive a reasonable and applicable initial stress field based on limited measurement results and engineering geological data to meet the needs of engineering^14–20^.

1. In the multiple linear regression method^21^, a numerical model based on the distribution of rock layers and topographic features is established. By considering gravity and several tectonic stress fields, a multiple linear regression equation is constructed. The solution of the equation gives regression factors for stress fields. The initial stress field is a linear combination of these stress fields multiplied by the corresponding regression factors^22–24^. This method is convenient and efficient, and the solution is unique, suitable for rock masses with simple geological conditions and has been widely used^25^. However, for engineering projects with complex geological conditions, in situ stress values obtained by the superposition of regression factors often differ greatly from the measured values. In addition, this method linearly superimposes different in situ stress fields, but in deep-buried underground engineering, the relationship between the in situ stress and burial depth is not linear.
2. The boundary load adjustment method^26,27^ repeatedly adjusts the calculated boundary load of the numerical model so that the calculated stress values under certain working conditions are close to the measured values. The initial in situ stress obtained by this method can be highly optimized for a specific space point but can hardly fit all measured points. In addition, there is no unique solution for this method, and the trial solution process has no rules to follow and can be time-consuming.
3. The displacement back analysis method^28^ uses the assumed in situ stress field and rock mass mechanical parameters to calculate the displacement of the engineering excavation through numerical simulation. The mechanical parameters of the rock mass are adjusted until the calculated displacement is consistent with the measured displacement. Then, the secondary stress field is obtained^29,30^. However, this method is only applicable to underground engineering during construction and cannot be applied in the engineering design stage^31^.
4. The valley evolution method^32,33^ uses layered excavation of ancient strata to simulate the formation process of the present topography and uses the lateral stress coefficient to indicate the variation in the stress field, thus obtaining the present in situ stress field. However, since large underground engineering stresses are affected by tectonic stress, it is difficult to determine the lateral stress coefficient, and because the lateral stress coefficient is not a constant value at various depths, this method can result in a large error in the initial in situ stress field inversion.
5. Artificial intelligence combined with the above methods^34–36^ can improve the efficiency and accuracy of in situ stress inversion. It can simulate the physiological mechanism of the human brain through a large number of neurons to realize intelligent information processing^37^. Artificial intelligence has remarkable advantages in nonlinear analysis and fuzzy recognition^38–40^. Commonly used artificial intelligence methods include neural networks^34^, genetic algorithms^41^, and gray theories^2^. These artificial intelligence methods usually aim at inversion of stress at measured points, not the global stress distribution, which results in good agreement at the measured points but uncertain accuracy elsewhere.

In this paper, a 3D numerical model of the Shuangjiangkou underground hydropower station in ancient times is established, which is used to invert initial in situ stress field. According to measured point data, the lateral stress coefficients of stress components in ancient times are estimated, and multiple combinations of stress components with different lateral stress coefficients are established by a uniform design test. Generative adversarial network (GAN) can automatically establish high-dimensional and global probability distribution of variables by confront training, and can generate new data samples according to the distribution of real data, which can improve the accuracy of rock mechanical parameters and models. Based on the nonlinear analysis of the GAN, two regression factors governing the distribution law of optimized lateral stress coefficients of each stress component in ancient times were determined. After GAN prediction of 12 regression factors for six stress components, optimized lateral stress coefficients are determined. By built model of ancient planation surface with no undulations, the ancient stress field can be reconstructed using the optimized lateral stress coefficients. Then, strata are excavated by layers to simulate valley evolution, and the final stress result represents the present in situ stress field and distribution law. Through comparison and verification with measurement points, this inversion method of initial in situ stress is found to be feasible and suitable for deep buried large underground engineering.

## GAN inversion method for initial in situ stress field

**Figure 1:**
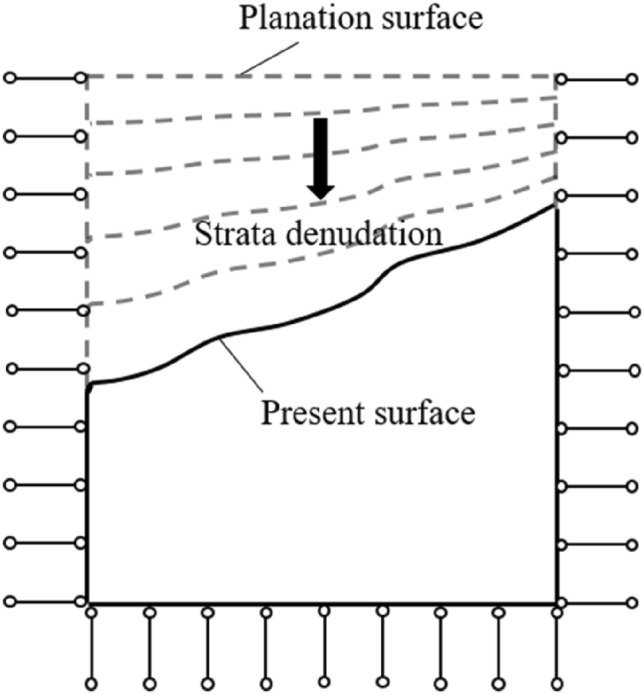
Schematic diagram of ancient strata denudation process.

The stress field in deep-cut valley area evolves from an ancient regional stress field, accompanied by river valley erosion, surface denudation and other geological effects to shape the valley slope in a long-term unloading process, prompting the rock mass to constantly adjust stress, strain and energy to form a new local stress field^42^. Therefore, top-down excavation of the ancient strata is used to simulate the gradual formation of the river valley geomorphic characteristics and the release of stress in the inversion of the present in situ stress field. The ancient strata denudation process is shown in Fig. 1, and the following assumptions are made about ancient surface and geological formations^43,44^.

1. The ancient surface is a planation surface with no obvious undulations.
2. The ancient stress field consists mainly of a self-gravity stress field and a tectonic stress field, and tectonic movement was completed in the ancient period.
3. The present in situ stress field evolved from an ancient stress field and was gradually formed by geological effects such as surface denudation and river erosion.

### Inversion of the ancient stress field based on the lateral stress coefficient

In recent years, a method using lateral stress coefficients to inverse in situ stress fields has been proposed and developed^32,33,42^ and is expressed as

1 $$\sigma_{i}^{{}} (x,y,z) = k_{i} \gamma H$$

where *σ*_1_–*σ*_6_ are stress components *σ*_*x*_*, σ*_*y*_*, σ*_*z*_*, τ*_*yz*_*, τ*_*zx*_ and *τ*_*xy*_. *k*_*i*_ is the lateral stress coefficient for the six stress components. *H* is burial depth. *γ* is bulk density.

However, the present stress field is affected by topographical fluctuations and rock mass mechanical properties, leading to the in situ stress coefficient varying greatly in different places^31^. In ancient times, the topography and geomorphology had small fluctuations, and the geological structure was simple. Therefore, utilizing lateral stress coefficients to invert the ancient stress field is more suitable.

Many scholars^25,37^ have considered that lateral stress coefficients are a certain value at each place in a rock mass, which means that stress components increase linearly as burial depth increases. However, for deep buried engineering, the lateral stress coefficient is found not to be linear with burial depth^45^. Based on global measured in situ stress data, Brown and Hoek^45^ found that the relationship between the lateral stress coefficient and the burial depth is

2 $$\frac{100}{H} + 0.3 \le \frac{{\sigma_{H} + \sigma_{h} }}{{2\sigma_{v} }} \le \frac{1500}{H} + 0.5$$

where *σ*_*H*_, *σ*_*h*_ and *σ*_*v*_ are the maximum principal stress, minimum principal stress on the horizontal plane and vertical stress, respectively.

Based on the statistical analysis of lateral stress coefficients of different rock masses in mainland China, Jing Feng^46^ reached conclusions similar to the ones of Eq. (2). Therefore, the relationship between the lateral stress coefficients and burial depth is

3 $$k_{i} = \frac{{a_{i} }}{H} + b_{i}$$

where *a*_*i*_ and *b*_*i*_ are regression factors.

Based on Eq. (3) and measured stresses in engineering, approximate lateral stress coefficients *k*_*i*_ of different stress components can be obtained. Introducing *k*_*i*_ to Eq. (1), an approximate ancient stress field can be obtained.

### Inversion of the present in situ stress field based on GAN

Based on the approximate ancient stress field, numerical simulation can be used to excavate the ancient strata layer by layer to obtain the present stress field. Since the ancient stress field is approximately estimated, there may be a large error in obtaining the present stress field. To solve this problem, this paper introduces the generative adversarial network (GAN) to fine-tune lateral stress coefficients in ancient times to optimize the present in situ stress field to meet the demand of engineering analysis.

**Figure 2:**
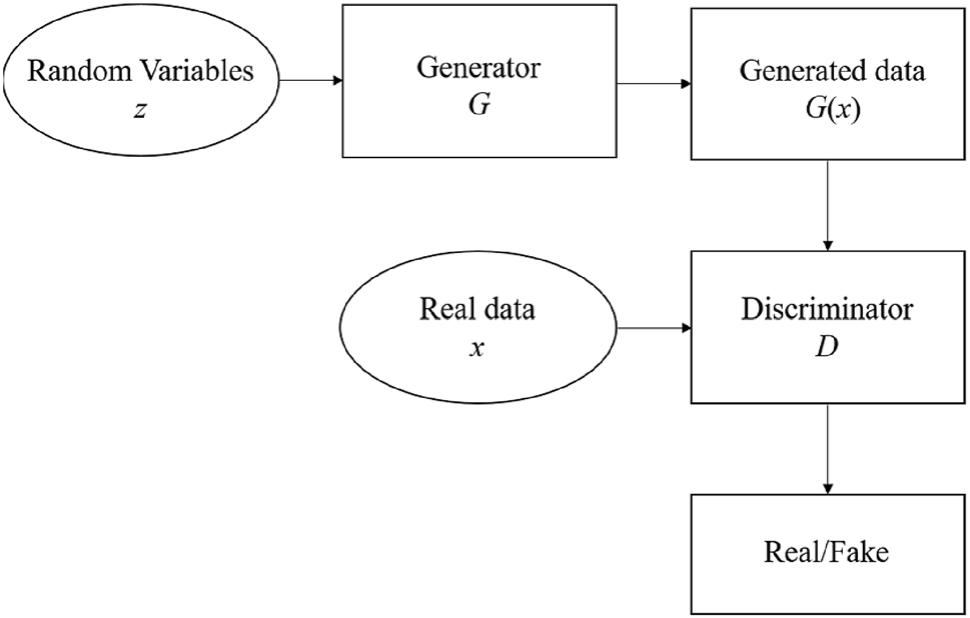
Calculation process of GAN.

GAN is a generative model proposed by Goodfellow et al.^47^, and further developed by Mirza et al.^48^, Odena et al.^49^ and Reed et al.^50^. Its basic thought is inspired by a minimax two-player game, and is widely used in artificial intelligence models, which show high learning and association capabilities and distributed processing capabilities to solve nonlinear problems. GAN consists of a generator and a discriminator trained on confrontational learning mechanism, as shown in Fig. 2. The purpose of GAN is to estimate the potential distribution of existing data and generate new data samples from the same distribution by a generator *G* which transforms the random variables *z* into the data that can be faked by continuously learning the probability distribution of real data. The discriminator *D* is a binary classifier that discriminates whether the input data is real or generated data samples. The two networks are enhanced simultaneously by competing with each other during training, so that the two networks constitute a dynamic game process, until Nash equilibrium^51^ is achieved. Both the generator and the discriminator can be designed in conjunction with the current deep neural networks^52^. The calculated process of GAN can be summarized as a binary minimax game, and the objective function can be defined as:

4 $$\mathop {\min }\limits_{G} \mathop {\max }\limits_{D} V(D,G) = E_{{x\sim P_{data(x)} }} [\lg D(x)] + E_{{z\sim P_{g(z)} }} [\lg (1 - D(G(z)))]$$

where *V*(*D*,*G*) is the cross-entropy loss of two classifications, *P*_*data*_(*x*) is the real data distribution, *P*_*g*_(*z*) is the random variables distribution, *G*(*z*) is samples generated by the generator based on the random variables, and *E*(⋅) means the calculated expected value. The global optimal solution is reached when *P*_*data*_ = *P*_*g*_. For GAN, the loss function of generator and discriminator are expressed as log(*D*(*G*(*z*))) and log(*D*(*x*)) + log(1 − *D*(*G*(*z*))).

In this paper, deep learning is carried out on the distribution of the lateral stress coefficient based on the superiority of GAN data enhancement. The basic process of using GAN to analyze the lateral stress coefficients is as follows.

1. From field survey data, the obtained stress values of the measured points are statistically analyzed, and the effective measured points are selected to determine the approximate lateral stress coefficients by Eq. (3).
2. Based on a uniform design test, multiple ancient stress fields with different values of *a*_*i*_ and *b*_*i*_ can be designed for a given floating range of *a*_*i*_ and *b*_*i*_ in Eq. (3).
3. According to multiple ancient stress fields, strata excavation is carried out using FLAC3D to obtain multiple present initial stress fields, from which lateral stress coefficients under the present burial depth are determined.
4. The coordinates of the measurement point, present burial depth, present lateral stress coefficient and regression factors in ancient times are regarded as real data sample. After data normalization, the data sample are input into the GAN for training.
5. After training in GAN, regression factors *a*_*i*_ and *b*_*i*_ of the optimized ancient lateral stress coefficients can be obtained. Then, the optimized ancient stress field is constructed by Eq. (1).
6. After excavating the optimized ancient stress field, the optimized present initial stress field can be obtained.

For the selection of *a*_*i*_ and *b*_*i*_ values, many scholars^25,53–56^ use orthogonal experiments. There are six pairs of regression factors *a*_*i*_ and *b*_*i*_ for six stress components. According to the principle of orthogonal experiments, (6 × 2)^2^ = 144 combinations need to be designed for numerical calculation, which can lead to an excessive number of orthogonal tests and is rather time-consuming. Therefore, it is more appropriate to use a uniform design test instead of an orthogonal test^22,57^. The advantage of the uniform design can greatly reduce the number of tests and keep training samples uniformly dispersed. In the uniform design test, multiple combinations with different values of *a*_*i*_ and *b*_*i*_ can be designed. The parameters of the measured points under different combinations of *a*_*i*_ and *b*_*i*_ are

5 $${\mathbf{x}} = \left[ {x_{1} ,x_{2} , \ldots x_{n} } \right]^{T}$$

6 $${\mathbf{y}} = \left[ {y_{1} ,y_{2} , \ldots y_{1n} } \right]^{T}$$

7 $${\mathbf{H}}^{{\mathbf{p}}} = \left[ {H_{1}^{p} ,H_{2}^{p} , \ldots H_{n}^{p} } \right]^{T}$$

8 $${\mathbf{k}}_{{\mathbf{j}}} = \left[ {\begin{array}{*{20}c} {k_{11} ,k_{12} ,k_{13} ,k_{14} ,k_{15} ,k_{16} } \\ {k_{21} ,k_{22} ,k_{23} ,k_{24} ,k_{25} ,k_{26} } \\ \vdots \\ {k_{{{\text{n}}1}} ,k_{{{\text{n}}2}} ,k_{{{\text{n}}3}} ,k_{{{\text{n}}4}} ,k_{{{\text{n}}5}} ,k_{{{\text{n}}6}} } \\ \end{array} } \right]$$

9 $${\mathbf{a}}_{{\mathbf{j}}} = \left[ {\begin{array}{*{20}c} {a_{11} ,a_{12} ,a_{13} ,a_{14} ,a_{15} ,a_{16} } \\ {a_{21} ,a_{22} ,a_{23} ,a_{24} ,a_{25} ,a_{26} } \\ \vdots \\ {a_{n1} ,a_{n2} ,a_{n3} ,a_{n4} ,a_{n5} ,a_{n6} } \\ \end{array} } \right]$$

10 $${\mathbf{b}}_{{\mathbf{j}}} = \left[ {\begin{array}{*{20}c} {b_{11} ,b_{12} ,b_{13} ,b_{14} ,b_{15} ,b_{16} } \\ {b_{21} ,b_{22} ,b_{23} ,b_{24} ,b_{25} ,b_{26} } \\ \vdots \\ {b_{n1} ,b_{n2} ,b_{n3} ,b_{n4} ,b_{n5} ,b_{n6} } \\ \end{array} } \right]$$

where **x** and **y** are the coordinate vectors in the horizontal plane of the measured points. *n* is the number of measured points. **H**^**p**^ is the burial depth vector of the measured points at present. **k**_**j**_ is the lateral stress coefficient matrix of the measured points at present and *j* is the number of uniform design tests. **a**_**j**_ and **b**_**j**_ are regression factors matrices of stress components *σ*_*x*_*, σ*_*y*_*, σ*_*z*_*, τ*_*yz*_*, τ*_*zx*_ and *τ*_*xy*_ of measured points in ancient times.

After each uniform design test, the training of the GAN can be carried out. The real data sample input to GAN can be expressed as:

11 $${\mathbf{P}} = \left[ {\begin{array}{*{20}c} {{\mathbf{x}},{\mathbf{y}},{\mathbf{H}}^{{\mathbf{p}}} ,{\mathbf{k}}_{{\mathbf{1}}} ,{\mathbf{a}}_{{\mathbf{1}}} ,{\mathbf{b}}_{{\mathbf{1}}} } \\ {{\mathbf{x}},{\mathbf{y}},{\mathbf{H}}^{{\mathbf{p}}} ,{\mathbf{k}}_{{\mathbf{2}}} ,{\mathbf{a}}_{{\mathbf{2}}} ,{\mathbf{b}}_{{\mathbf{2}}} } \\ \vdots \\ {{\mathbf{x}},{\mathbf{y}},{\mathbf{H}}^{{\mathbf{p}}} ,{\mathbf{k}}_{{\mathbf{j}}} ,{\mathbf{a}}_{{\mathbf{j}}} ,{\mathbf{b}}_{{\mathbf{j}}} } \\ \end{array} } \right]$$

After training, input the data of measured points to GAN, the optimized *a*_*i*_ and *b*_*i*_ values can be obtained. The optimized ancient stress field can be calculated by Eq. (3), and the present stress field can then be obtained after a nonlinear elastic–plastic excavation simulation.

### Equivalent mechanical parameters of faults

The parameters between the fault and the rock mass are quite different. If the influence of the fault is not considered in the inversion of the present stress field, it causes a large error in the result^58^. Therefore, the parameters of faults should be assigned to the corresponding element before simulating the excavation of ancient strata. Faults and underground caverns do not usually intersect in an orthogonal form and are often simplified into thin elements in numerical simulation analysis. This method of treating faults may bring difficulties in the mesh generation of models due to the limited thickness and complicated intersection relationship of faults.

**Figure 3:**
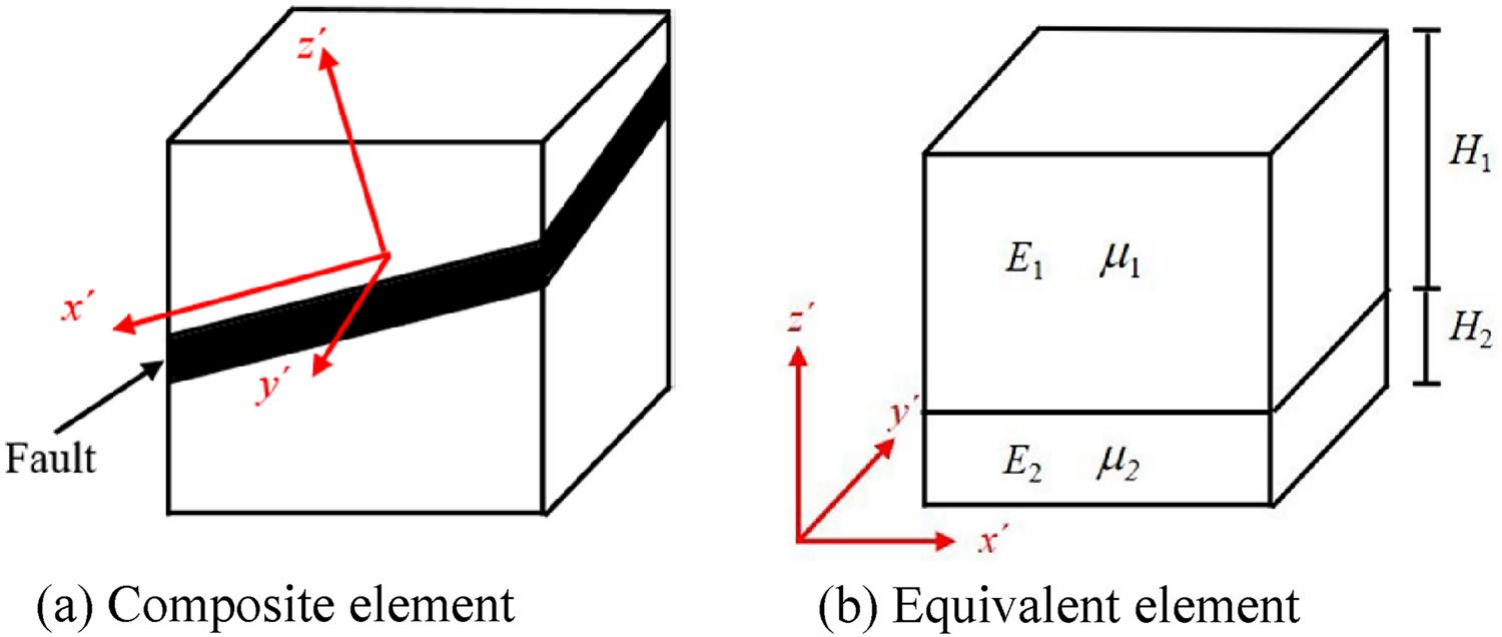
Schematic diagram of fault cutting rock mass element.

In this paper, the rock mass is divided into hexahedron elements. Due to the existence of faults, some rock mass elements are cut by faults. These elements contain both rock masses and faults, forming composite elements, as shown in Fig. 3a. For one element, only a set of mechanical parameters can be assigned, so the mechanical parameters of composite elements with both rock masses and faults need to be equalized^59^. It is assumed that the composite element is a transversely isotropic material in the local coordinate system (*x*′*y*′*z*′) established on the fault plane. The composite element is simplified into an equivalent element distributed in layers, and the parameters are shown in Fig. 3b. *H*_*k*_ is the layer thickness, which can be expressed as

12 $$H_{k} = V_{k} /A$$

where subscript *k* equals 1 or 2, representing the parameters of the rock mass and fault, respectively. *V*_*k*_ is volume of rock mass or fault. *A* is the area of the contact surface between the rock mass and the fault.

For the *z*′ direction, the deformation and stress of the equivalent element are

13 $$\left\{ {\begin{array}{*{20}l} {\overline{\sigma }_{v} = \sigma_{v1} = \sigma_{v2} } \hfill \\ {\Delta l = \frac{{\sigma_{v1} }}{{E_{1} }}H_{1} + \frac{{\sigma_{v2} }}{{E_{2} }}H_{2} = \frac{{\sigma_{v} }}{{\overline{E}_{v} }}(H_{1} + H_{2} )} \hfill \\ \end{array} } \right.$$

where ${\tilde{\sigma }}_{v}$, *σ*_*v*1_ and *σ*_*v*2_ are the stress applied in the equivalent element, rock mass and fault, respectively, in the *z*′ direction. ${\overline{E} }_{v}$ is the equivalent elastic modulus in *z*′ direction.

Based on Eq. (13), the equivalent elastic modulus in the *z*′ direction is

14 $$\overline{E}_{v} = {{(H_{1} + H_{2} )} \mathord{\left/ {\vphantom {{(H_{1} + H_{2} )} {\left( {\frac{{H_{1} }}{{E_{1} }} + \frac{{H_{2} }}{{E_{2} }}} \right)}}} \right. \kern-\nulldelimiterspace} {\left( {\frac{{H_{1} }}{{E_{1} }} + \frac{{H_{2} }}{{E_{2} }}} \right)}}$$

For the *x*′ and *y*′ directions, assuming that the elongation of the rock mass and fault in the equivalent element are equal, then

15 $$\left\{ {\begin{array}{*{20}l} {\overline{\varepsilon }_{h} = \varepsilon_{h1} = \varepsilon_{h2} } \hfill \\ {\overline{\sigma }_{h} (H_{1} + H_{2} ) = \sigma_{h1} H_{1} + \sigma_{h2} H_{2} } \hfill \\ \end{array} } \right.$$

where ${\overline{\varepsilon }}_{h}$, *ε*_*h*1_ and *ε*_*h*2_ are the strain in the equivalent element, rock mass and fault in the *x*′ and *y*′ directions, respectively. ${\overline{\sigma }}_{h}$, *σ*_*h*1_ and *σ*_*h*2_ are the stresses applied in the equivalent element, rock mass and fault in the *x*′ and *y*′ directions, respectively.

Equation (15) can be written as

16 $$\overline{E}_{h} \overline{\varepsilon }_{h} (H_{1} + H_{2} ) = E_{1} \varepsilon_{h1} H_{1} + E_{2} \varepsilon_{h2} H_{2}$$

where ${\overline{E} }_{h}$ is the equivalent elastic modulus in the *x*′ and *y*′ directions.

Based on Eqs. (15) and (16), the equivalent elastic modulus is

17 $$\overline{E}_{h} = \frac{{E_{1} H_{1} + E_{2} H_{2} }}{{H_{1} + H_{2} }}$$

Combining Eqs. (15) and (17), the equivalent Poisson’s ratio $\overline{\mu }$ can be expressed as

18 $$\begin{aligned} \overline{\mu } & = \frac{{\overline{\varepsilon }_{h} }}{{\overline{\varepsilon }_{v} }} = \frac{{\overline{\sigma }_{h} }}{{\overline{E}_{h} }} \cdot \frac{1}{{\overline{\varepsilon }_{v} }} = \frac{{\sigma_{h1} H_{1} + \sigma_{h2} H_{2} }}{{\overline{E}_{h} (H_{1} + H_{2} )}} \cdot \frac{1}{{\overline{\varepsilon }_{v} }} \\ & = \frac{{\mu_{1} E_{1} \varepsilon_{v1} H_{1} + \mu_{2} E_{2} \varepsilon_{v2} H_{2} }}{{\overline{E}_{h} (H_{1} + H_{2} )}} \cdot \frac{1}{{\overline{\varepsilon }_{v} }} \\ & = \frac{{\mu_{1} \sigma_{v1} H_{1} + \mu_{2} \sigma_{v2} H_{2} }}{{\overline{E}_{h} (H_{1} + H_{2} )}} \cdot \frac{{\overline{E}_{v} }}{{\overline{\sigma }_{v} }} \\ & = \frac{{\mu_{1} H_{1} + \mu_{2} H_{2} }}{{H_{1} + H_{2} }} \cdot \frac{{\overline{E}_{v} }}{{\overline{E}_{h} }} \\ \end{aligned}$$

## Project overview and in situ stress measurement analysis

**Figure 4:**
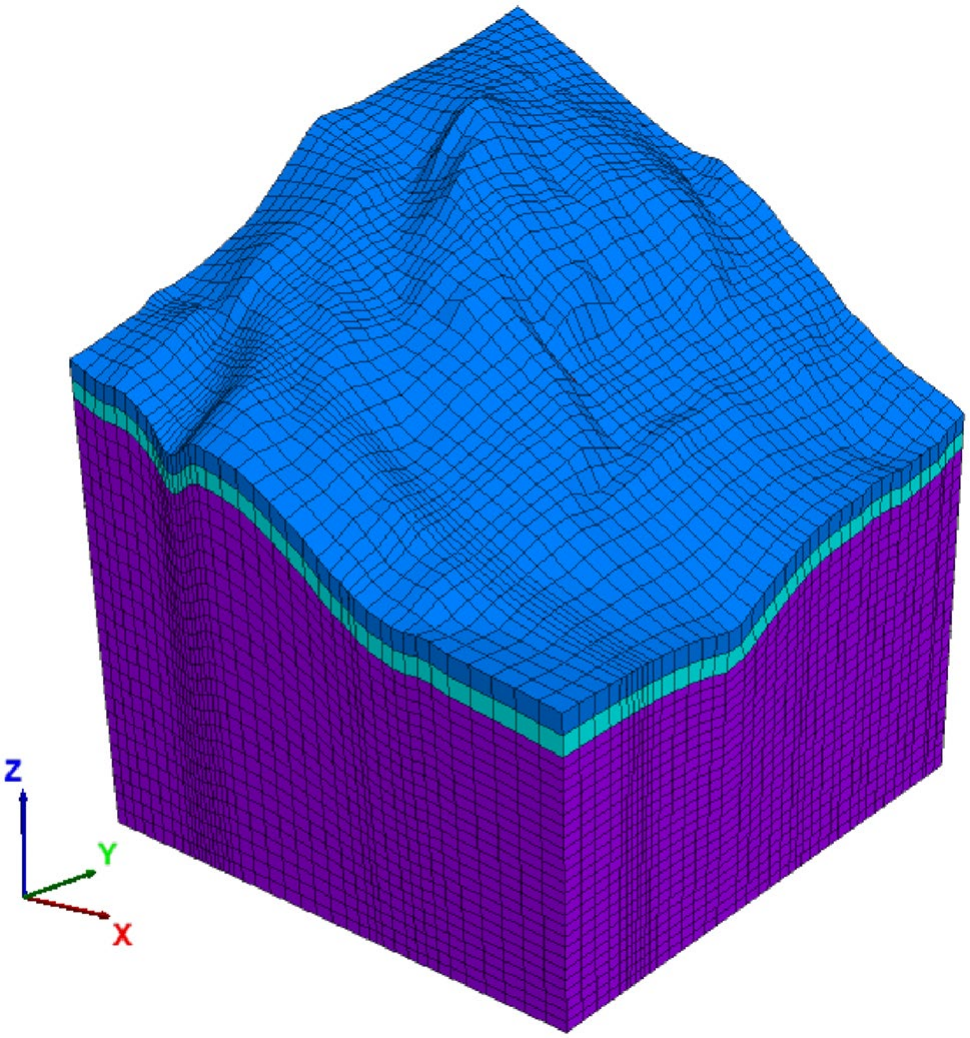
3D model of the mountain where the Shuangjiangkou hydropower station located.

**Figure 5:**
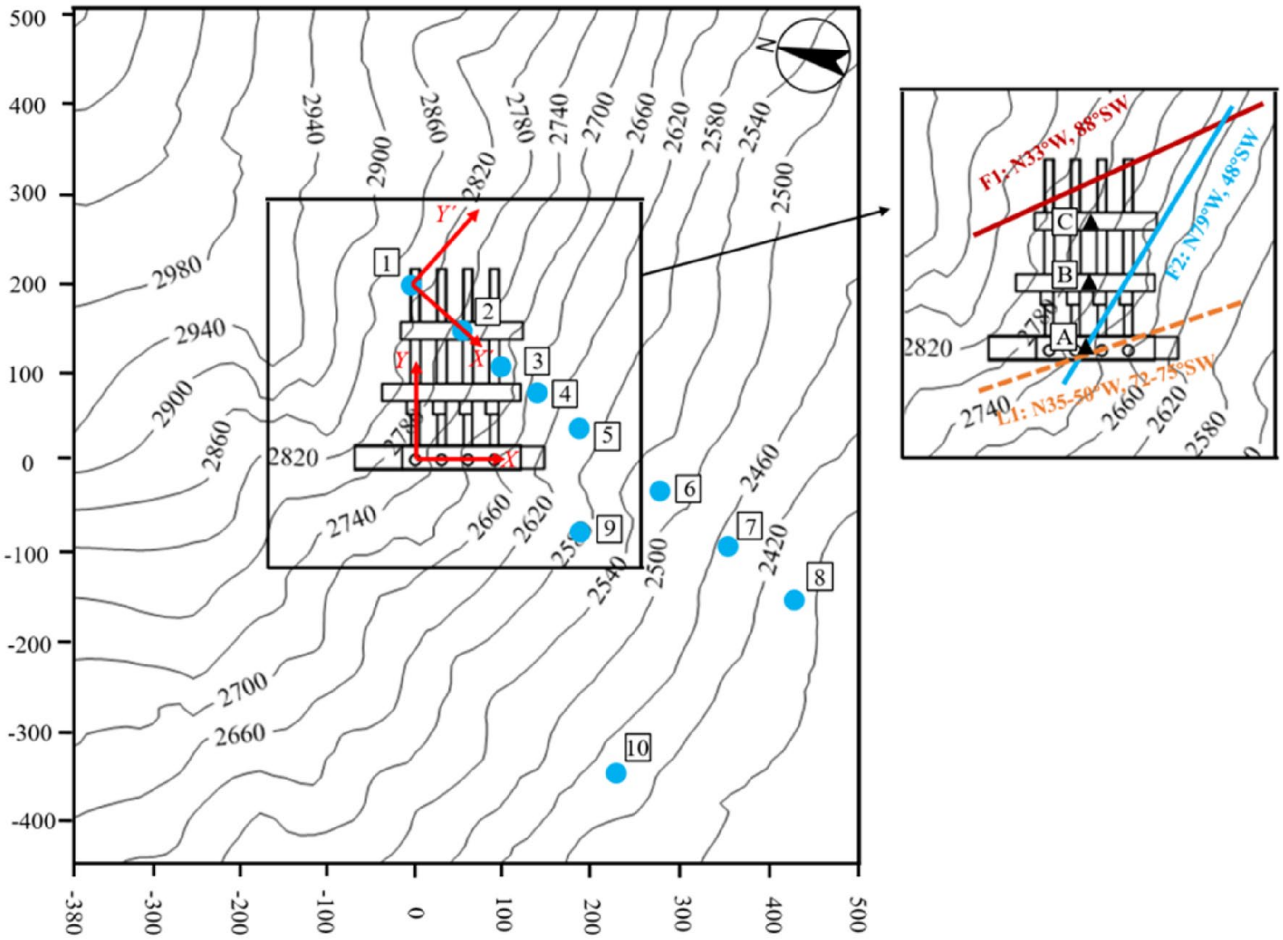
Schematic diagram of the underground powerhouses, faults, and measured points.

The Shuangjiangkou hydropower station is located on the Dadu River in western Sichuan Province, China. The diversion and power generation structures of the hydropower station are arranged on the left bank of the mountain. The length of the 3D numerical model is 878.14 m in the *X* direction and 950 m in the *Y* direction, as shown in Fig. 4. The *X* direction is N170° E along the axis of the main powerhouse. The length of the main powerhouse in the underground powerhouse is 217.5 m, the arch span is 28.3 m, the maximum excavation depth is 68.3 m, the horizontal burial depth is 400–640 m, and the vertical burial depth is 320–500 m. Two faults F1 and F2 and a lamprophyre L1 are found in the engineering site, as shown in Fig. 5.

The distribution of the ten measured points is shown in Fig. 5, and the measured in situ stress data are shown in Table 1. As the measured in situ stress is obtained in the geodetic coordinate system, it needs to be converted to a model coordinate system, as shown in Table 2. The measured in situ stress in the underground powerhouse has the following distribution characteristics.

1. The maximum principal stress *σ*_1_ is 14.88–28.96 MPa, the intermediate principal stress *σ*_2_ is 8.53–20.37 MPa, and the minimum principal stress *σ*_3_ is 3.14–10.88 MPa, indicating that the rock mass is in a triaxially unequal pressure state.
2. The average values of *σ*_*x*_/*σ*_*z*_ and *σ*_*y*_/*σ*_*z*_ are 1.44 and 1.22, respectively, indicating that the measured values of in situ stress show *σ*_*x*_ > *σ*_*y*_ > *σ*_*z*_. However, *σ*_*y*_/*σ*_*z*_ of point 5 is significantly less than average.
3. The average density *ρ* and Poisson's ratio *μ* of the rock mass are 2600 kg/m^3^ and 0.3, respectively, and the lateral stress coefficient *λ* = *μ*/(1 − *μ*) is 0.4286. The average values of *σ*_*x*_/*λσ*_*z*_ and *σ*_*y*_/*λσ*_*z*_ are 3.3559 and 2.8433, respectively, which are greater than 1.0. This indicates that the horizontal stress component in the measured stress is larger than the horizontal stress formed by Poisson’s ratio effect of the rock mass gravity. Therefore, there is a horizontal tectonic stress field in this area.
4. *σ*_*x*_/*σ*_*y*_ is greater than 1.00, indicating that the horizontal tectonic stress in the *Y* direction is smaller than that in the *X* direction.
5. *σ*_*z*_/*ρgh* is the ratio of *σ*_*z*_ at a point to the vertical stress formed by gravity of the overlying rock mass. Except for at the measured point 3, *σ*_*z*_*/ρgh* is 1.08–3.06, and the average value is 1.97.

**Table 1:** In situ stress parameters measured.

Point	Altitude (m)	Depth (m)	*σ*_1_			*σ*_2_			*σ*_3_		
			Magnitude (MPa)	Direction (°)	Dip angle (°)	Magnitude (MPa)	Direction (°)	Dip angle (°)	Magnitude (MPa)	Direction (°)	Dip angle (°)
1	2268	549	24.56	349.0	18.0	20.37	92.0	35	10.52	237.0	49.0
2	2268	470	28.96	325.0	27.2	18.83	72.5	30.3	10.88	201.4	47.0
3	2268	431	16.91	357.0	19.0	10.32	92.0	14.0	8.01	216.0	66.0
4	2268	357	27.29	310.4	− 3.5	18.27	36.8	45.6	8.49	223.8	44.2
5	2268	308	37.82	331.6	46.8	16.05	54.1	− 7.0	8.21	137.7	42.3
6	2268	238	19.21	323.0	− 3.5	13.61	49.2	8.6	5.57	300.4	64.8
7	2268	173	22.11	332.0	30.1	11.63	84.0	32.9	5.86	210.1	42.3
8	2268	107	15.98	325.6	30.1	8.53	81.8	37.3	3.14	208.5	38.1
9	2405	185	15.88	78.3	23.8	10.38	335.5	26.7	5.14	203.9	52.8
10	2272	183	14.88	30.6	23.2	10.19	138.8	36.2	7.50	275.4	44.8

Positive normal stress indicates compression and negative normal stress indicates tension.

**Table 2:** In situ stress components (MPa).

Point	*σ*_*x*_	*σ*_*y*_	*σ*_*z*_	*τ*_*yz*_	*τ*_*xz*_	*τ*_*xy*_
1	23.490	16.921	15.039	4.441	− 3.192	1.539
2	22.743	19.263	16.663	0.329	− 7.109	4.708
3	15.953	10.206	9.081	0.863	− 2.595	− 0.515
4	21.855	18.641	13.554	4.293	− 2.465	6.811
5	22.183	15.843	24.054	− 5.520	− 3.605	1.124
6	16.739	13.732	7.919	3.283	3.838	1.181
7	16.884	11.069	11.647	0.448	− 6.522	3.855
8	11.120	8.191	8.347	0.306	− 4.991	3.721
9	9.071	14.386	7.943	3.437	− 2.153	0.744
10	12.368	10.614	9.587	2.407	− 0.933	− 2.304

Point 3 and point 5 are relatively near Fault 2 and the lamprophyre, respectively, which may cause undulation of the measured value^10^. Therefore, in the inversion of the initial in situ stress, points 3 and 5 are not used.

## Inversion and analysis of stress field

### Approximate stress field

**Figure 6:**
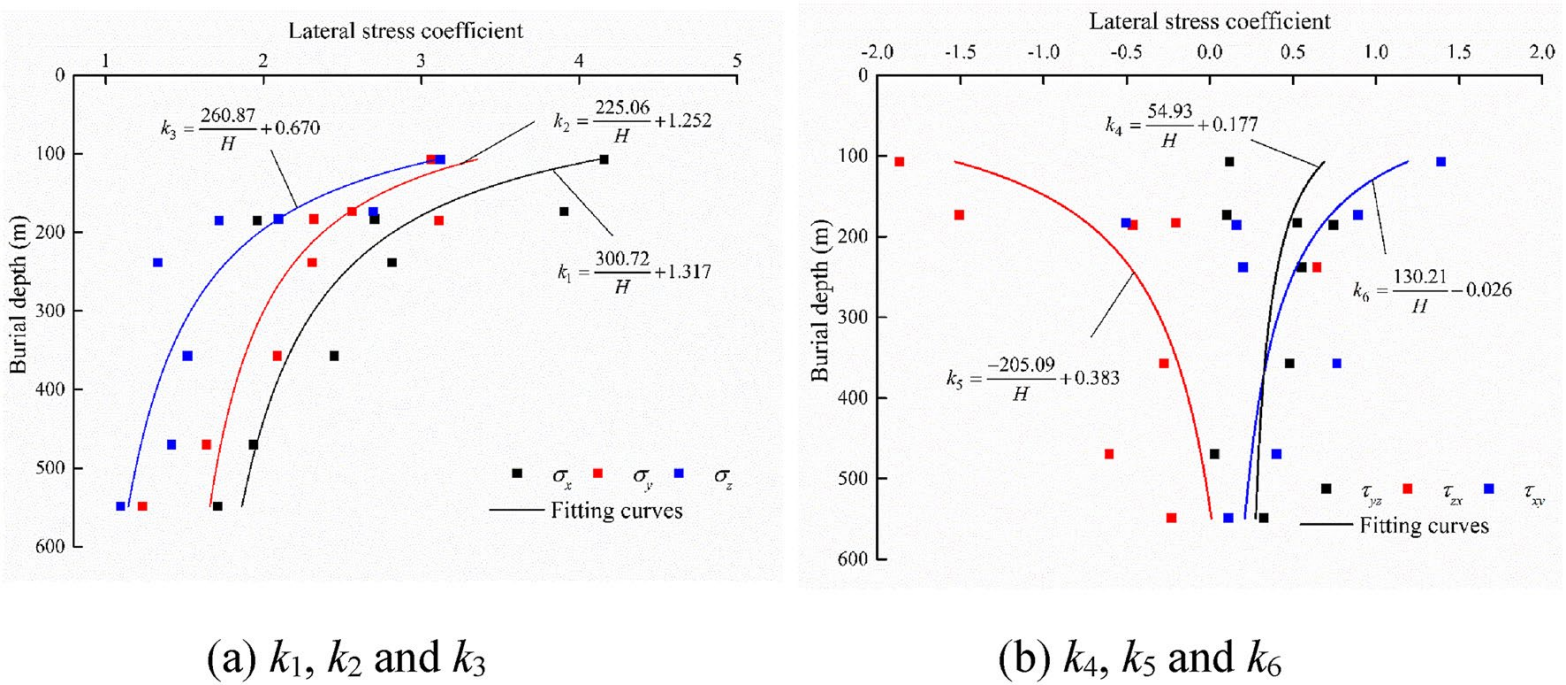
Relationship between burial depth and *k*_*i*_*.*

**Figure 7:**
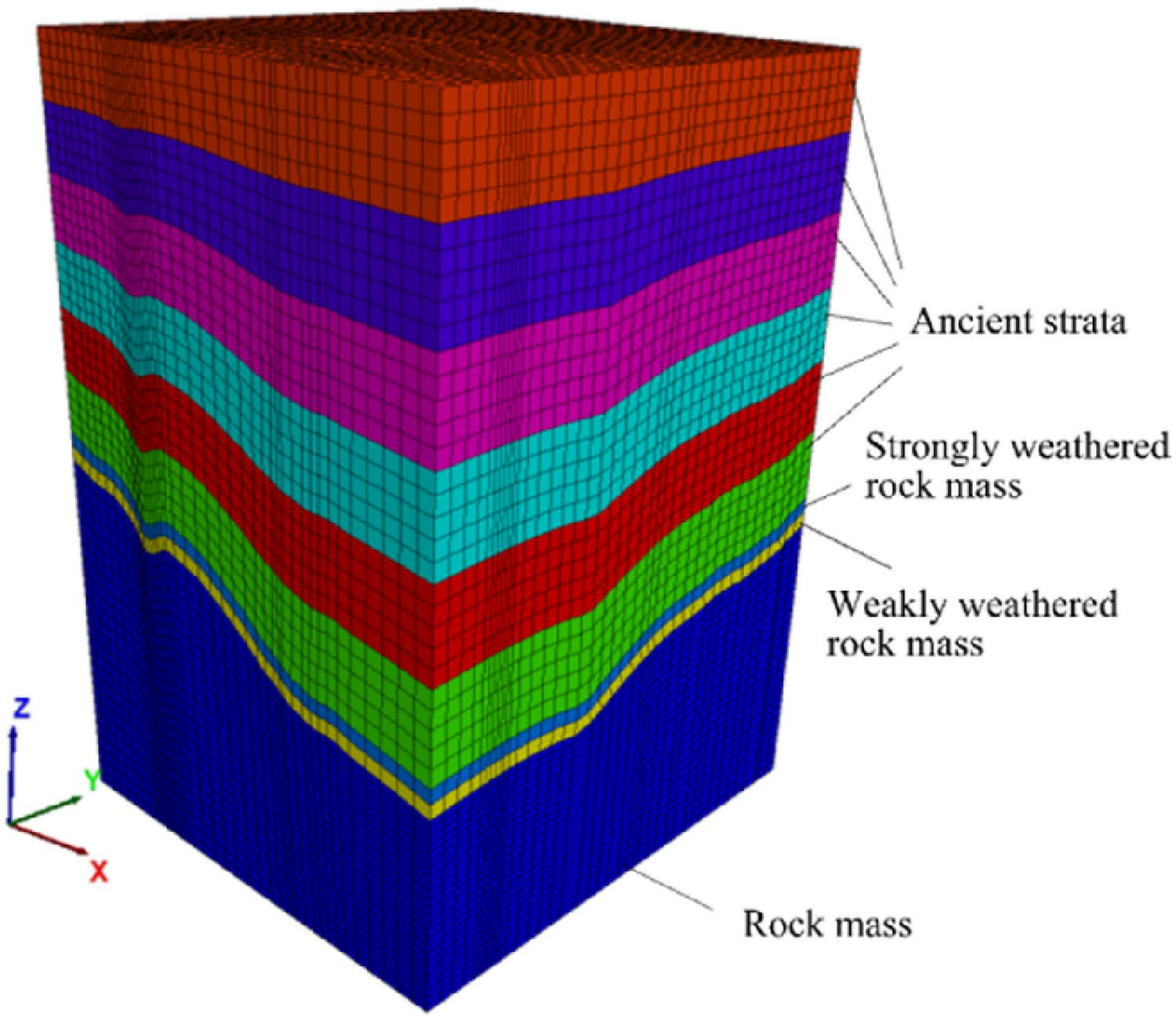
The ancient model.

By the analysis of the measured in situ stress in “Project overview and in situ stress measurement analysis”, except for measured points 3 and 5, other measured in situ stress points are considered to be representative sample points. In Table 3, the actual lateral stress coefficients *k*_*i*_ for the six stress components can be calculated. Based on Eq. (3), the relationship between the present burial depth and *k*_*i*_ is shown in Fig. 6. The fitted stress function can be imported into FLAC3D to form an approximate ancient stress field. As shown in Fig. 7, the *Z* direction range of the ancient model is from the bottom elevation of 1970 m to the planation plane. The upper six layers in the model are elements for excavation, which are used to simulate the denudation process of the valley. The parameters input to the ancient model in FLAC3D are listed in Table 4. The coordinates of elements containing both rock masses and faults are exported by the FISH language. Then, the parameters of equivalent elements are calculated based on “Equivalent mechanical parameters of faults” and assigned to the model. The equivalent element is calculated using a transversely isotropic elastic constitutive model.

**Table 3:** Lateral stress coefficient *k*_*i*_ for six stress components.

Point	*k*_1_	*k*_2_	*k*_3_	*k*_4_	*k*_5_	*k*_6_
1	1.7122	1.2334	1.0962	0.3237	− 0.2327	0.1122
2	1.9363	1.6401	1.4187	0.0280	− 0.6053	0.4008
4	2.4497	2.0895	1.5193	0.4812	− 0.2763	0.7634
6	2.8144	2.3088	1.3315	0.5520	0.6453	0.1986
7	3.9054	2.5603	2.6940	0.1036	− 1.5086	0.8917
8	4.1587	3.0633	3.1216	0.1144	− 1.8665	1.3916
9	1.9621	3.1117	1.7181	0.7434	− 0.4657	0.1609
10	2.7045	2.3209	2.0964	0.5263	− 0.2040	− 0.5038

**Table 4:** The parameters for numerical simulations.

Type of rock mass	Bulk density *γ* (kN/m^3^)	Deformation modulus *E* (GPa)	Poisson ratio *μ*	Internal friction coefficient *f*	Cohesion *c* (MPa)
Unweathered	26.0	12.5	0.30	1.25	1.55
Weakly weathered	25.5	7.0	0.32	0.95	0.90
Strongly weathered	23.5	3.0	0.35	0.70	0.40
Weak structural plane	18.0	0.3	0.38	0.40	0.03

In FLAC3D, each element zone stores six stress components, and each element grid point stores unbalanced forces. If unbalanced forces in the *X*, *Y*, *Z* directions are not applied to model element grid points, stresses in element zones change, and the calculated results of the model greatly deviate from the real results. To optimize the model in subsequent calculations, based on guidelines of the FLAC3D user manual, the model is performed as follows^60^ to perform ground stress balance.

1. Based on Eq. (3), ancient stress field can be calculated and imported to the ancient model in FLAC3D, then the parameters of the rock mass are input to the model.
2. The boundary normal velocities of the ancient model periphery and bottom surfaces are fixed to zero.
3. Calculate the model for one step, that is, run the *step 1* command to solve the model, and then record the unbalanced forces on element grid points.
4. Apply forces opposite to unbalanced forces recorded in (2) to each element grid point in the ancient model.
5. Repeat (3) and (4) several times to make the stress components close enough to the stress components in the ancient model.

After the above process, the balance calculation of the approximate ancient field is completed. Excavating ancient strata layer by layer can obtain the present in situ stress field. Six stress components and lateral stress coefficients at the measured points can be obtained. However, these lateral stress coefficients are obtained by approximating the ancient stress field, so there may be some unacceptable errors, and further optimized regression factors need to be determined.

### Optimized stress field

By using a GAN to inversely optimize the present stress field, it is necessary to construct adequate real samples for training. Since the initial in situ stress inversion of this project is to provide accurate ground stress for the underground cavern excavation, the measured points in and around the underground cavern need to be selected as samples. In addition, boundary effects occur in numerical excavation simulations, so measured points near the boundary of the numerical model need to be selected as samples. Therefore, the measured point in Table 3 can be as samples to GAN training. Based on a large number of trial calculations^25^, the floating range of regression factors in Eq. (3) is determined. The value range of each regression factor *a*_*i*_ or *b*_*i*_ is between 0.5 and 1.5 times of itself. Twelve regression factors are taken as factors of the uniform design test, and different values of regression factors are regarded as different levels. Based on the principle of uniform design, there are 13 levels for regression factors. Then, levels 1–13 correspond to regression factors 0.52, 0.60, 0.68, 0.76, 0.84, 0.92, 1.00, 1.08, 1.16, 1.24, 1.32, 1.40, and 1.48 times, respectively, so the U_13_ uniform table is selected^56^, as shown in Table 5. Regression factors of each test can be taken into Eqs. (1) and (3) to calculate the stress components of each element, which can be imported into FLAC3D for calculation. In each test, the calculated stress components and lateral stress coefficients of the measured points can be obtained. There are eight effective measured points, so eight training samples can be created in each test, for a total of 13 × 8 = 104 samples.

**Table 5:** U_13_ uniform table of levels combination of different factors in each test.

Test no.	Levels of different factors											
	*a*_1_	*b*_1_	*a*_2_	*b*_2_	*a*_3_	*b*_3_	*a*_4_	*b*_4_	*a*_5_	*b*_5_	*a*_6_	*b*_6_
1	156.37	0.790	153.04	0.952	219.13	0.616	54.93	0.191	− 237.90	0.475	171.88	− 0.036
2	180.43	1.001	207.06	1.352	323.48	0.938	28.56	0.120	− 172.28	0.383	151.04	− 0.034
3	204.49	1.212	261.07	1.753	156.52	0.563	59.32	0.234	− 106.65	0.291	130.21	− 0.032
4	228.55	1.422	315.08	0.851	260.87	0.884	32.96	0.163	− 254.31	0.199	109.38	− 0.030
5	252.60	1.633	135.04	1.252	365.22	0.509	63.72	0.092	− 188.68	0.506	88.54	− 0.028
6	276.66	1.844	189.05	1.653	198.26	0.831	37.35	0.205	− 123.05	0.414	67.71	− 0.026
7	300.72	0.685	243.06	0.751	302.61	0.456	68.11	0.135	− 270.72	0.322	182.29	− 0.024
8	324.78	0.896	297.08	1.152	135.65	0.777	41.75	0.248	− 205.09	0.230	161.46	− 0.022
9	348.84	1.106	117.03	1.552	240.00	0.402	72.51	0.177	− 139.46	0.536	140.63	− 0.020
10	372.89	1.317	171.05	0.651	344.35	0.724	46.14	0.106	− 287.13	0.444	119.79	− 0.018
11	396.95	1.528	225.06	1.052	177.39	0.348	76.90	0.219	− 221.50	0.352	98.96	− 0.016
12	421.01	1.738	279.07	1.452	281.74	0.670	50.54	0.149	− 155.87	0.260	78.13	− 0.014
13	445.07	1.949	333.09	1.853	386.09	0.992	81.30	0.262	− 303.53	0.567	192.71	− 0.038

**Figure 8:**
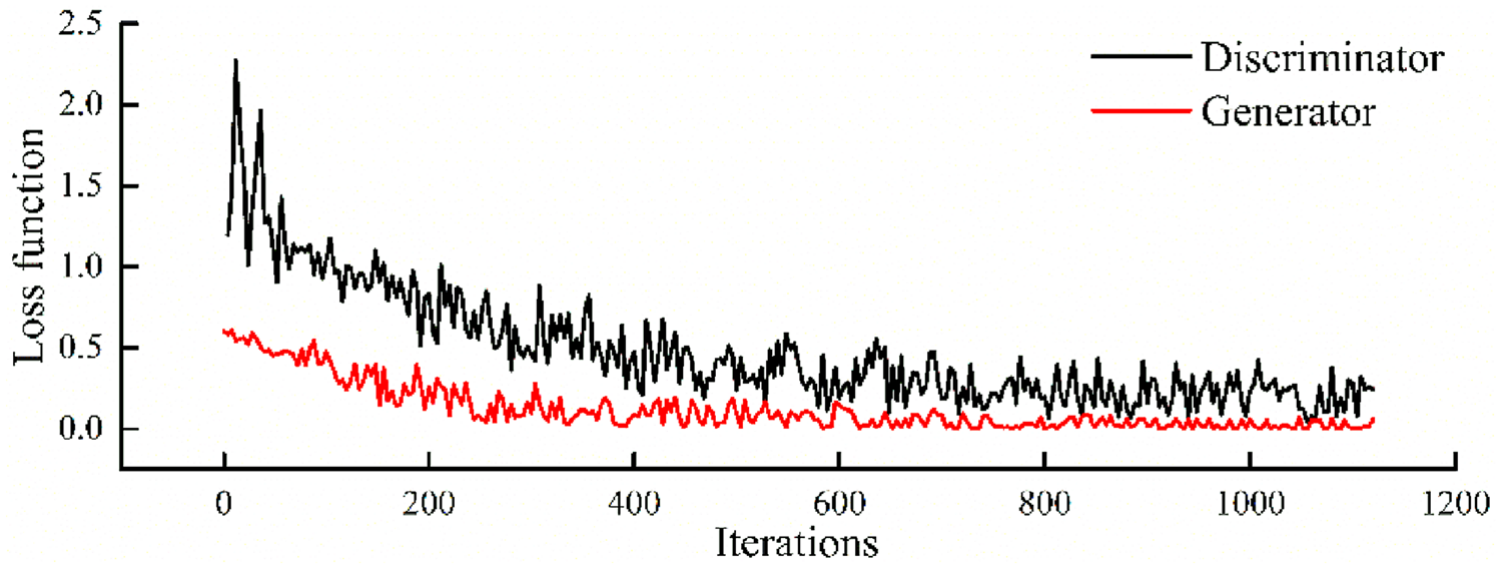
The loss function of GAN.

After training with real data samples, input *x* and *y* coordinates, present burial depth and lateral stress coefficient of the measured points in the present stress field of measured points, the regression factors of the ancient stress field can be obtained. Then, the optimized ancient stress field can be obtained by Eq. (3). Figure 8 shows the relationship between the loss function of generator and discriminator in GAN and iterations. The generator *G* and the discriminator network *D* are continuously confronted, so that the loss function of the two network reduces to a minimum, indicating that the output sample data from generator is stable. After GAN training, the regression factors of the optimized ancient stress field can be predicted, so the optimized ancient stress field can be obtained by Eq. (3). After excavating the optimized ancient stress field, the present stress field can be obtained. The regression factors and calculated stress values of the measured points are listed in Tables 6 and 7. The relative error Δ of the measured points can be calculated:

19 $$\Delta = \frac{{\left\| {\hat{\sigma }_{i} - \sigma_{i} } \right\|_{2} }}{{\left\| {\sigma_{i} } \right\|_{2} }} \times 100\%$$

where $\hat{\sigma }_{i}$ is the calculated stress component and $\sigma_{i}$ is the measured stress component. $\left\| \cdots \right\|_{2}$ is the 2-Norm.

**Table 6:** Regression factors in ancient stress field obtained by GAN and BP neutral network.

	Regression factor of stress components					
	*σ*_*x*_	*σ*_*y*_	*σ*_*z*_	*τ*_*yz*_	*τ*_*xz*_	*τ*_*xy*_
**GAN**						
*a*_*i*_	174.58	165.36	395.52	45.63	− 215.36	139.10
*b*_*i*_	1.236	0.801	0.526	0.365	0.326	0.003
**BP neural network**						
*a*_*i*_	185.30	160.69	382.25	48.65	− 201.36	139.45
*b*_*i*_	0.999	0.833	0.610	0.224	0.220	− 0.016

**Table 7:** Stress component values of each measured point based on GAN and BP neutral network.

Point	*σ*_*x*_ (MPa)	*σ*_*y*_ (MPa)	*σ*_*z*_ (MPa)	*τ*_*yz*_ (MPa)	*τ*_*xz*_ (MPa)	*τ*_*xy*_ (MPa)	Relative error (%)
**1**							
Measured	23.490	16.921	15.039	4.441	− 3.192	1.539	
GAN	25.153	18.653	16.053	5.012	− 3.036	1.754	8.10
BP	25.632	18.312	15.851	5.265	− 2.880	2.215	8.76
**2**							
Measured	22.743	19.263	16.663	0.329	− 7.109	4.708	
GAN	23.965	17.523	15.056	1.253	− 6.326	3.215	9.34
BP	24.262	17.286	15.116	1.189	− 5.955	3.240	10.18
**4**							
Measured	21.855	18.641	13.554	4.293	− 2.465	6.811	
GAN	22.365	15.213	14.245	5.213	− 2.523	5.036	12.36
BP	21.689	15.623	13.901	5.132	− 2.250	5.189	10.82
**6**							
Measured	16.739	13.732	7.919	3.283	3.838	1.181	
GAN	17.523	13.201	10.653	4.456	1.253	1.956	17.46
BP	17.813	13.333	11.442	4.779	− 0.842	2.004	25.58
**7**							
Measured	16.884	11.069	11.647	0.448	− 6.522	3.855	
GAN	15.523	12.236	11.985	0.523	− 4.985	3.562	9.81
BP	15.621	12.172	10.605	0.884	− 5.853	2.913	9.50
**8**							
Measured	11.120	8.191	8.347	0.306	− 4.991	3.721	
GAN	11.735	10.32	8.563	0.521	− 3.652	3.263	15.30
BP	11.735	11.680	9.762	1.723	− 2.315	3.073	28.40
**9**							
Measured	9.071	14.386	7.943	3.437	− 2.153	0.744	
GAN	8.896	16.236	7.523	3.236	− 2.475	0.856	10.12
BP	9.374	15.816	8.154	4.263	− 3.002	0.594	9.88
**10**							
Measured	12.368	10.614	9.587	2.407	− 0.933	− 2.304	
GAN	12.956	10.985	10.036	2.563	− 0.902	− 1.365	6.56
BP	13.751	11.448	10.308	2.154	− 1.368	0.216	16.23

For comparison, regression factor of stress components obtained by GAN and BP neural network are also listed in Tables 6 and 7. For these method, the relative errors of *σ*_*x*_, *σ*_*y*_ and *σ*_*z*_ are less than 15%, which is more optimized than *τ*_*yz*_, *τ*_*xz*_ and *τ*_*xy*_. According to statistical data, the error of in situ stress measurement results can reach 25–30%^61^. The maximum relative error of the measured points with GAN is 17.46%, so the distribution law of the in situ stress field is reasonable. For BP method, the maximum relative error for Point 8 reaches 28.4%, so the average relative error of GAN is much smaller than that of BP neural network, especially for the inversion of *τ*_*yz*_, *τ*_*xz*_ and *τ*_*xy*_.

### Analysis of present in situ stress field based on GAN inversion method

**Figure 9:**
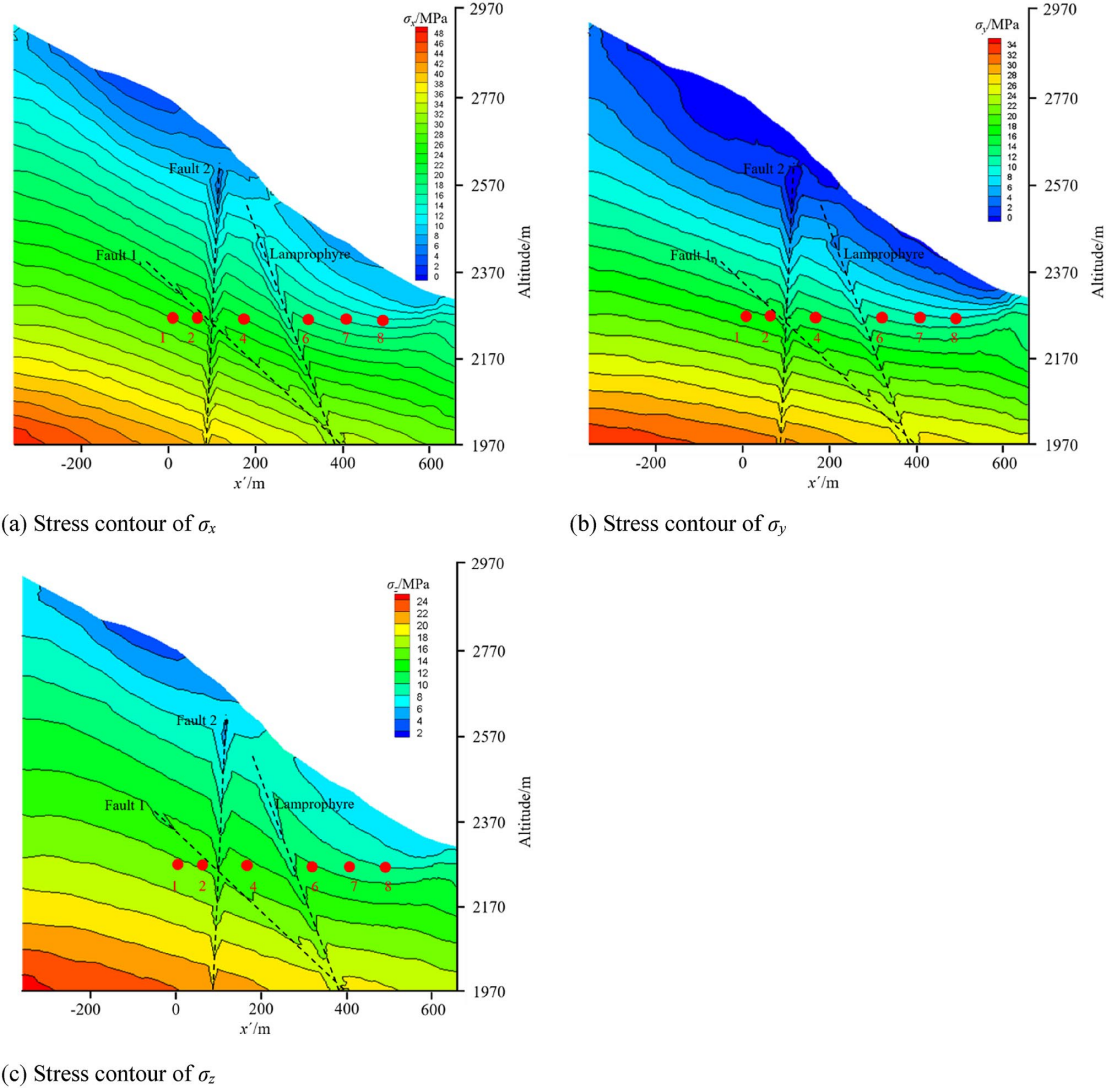
Stress contour of *σ*_*x*_, *σ*_*y*_, *σ*_*z*_ at *y*′ = 0.

**Figure 10:**
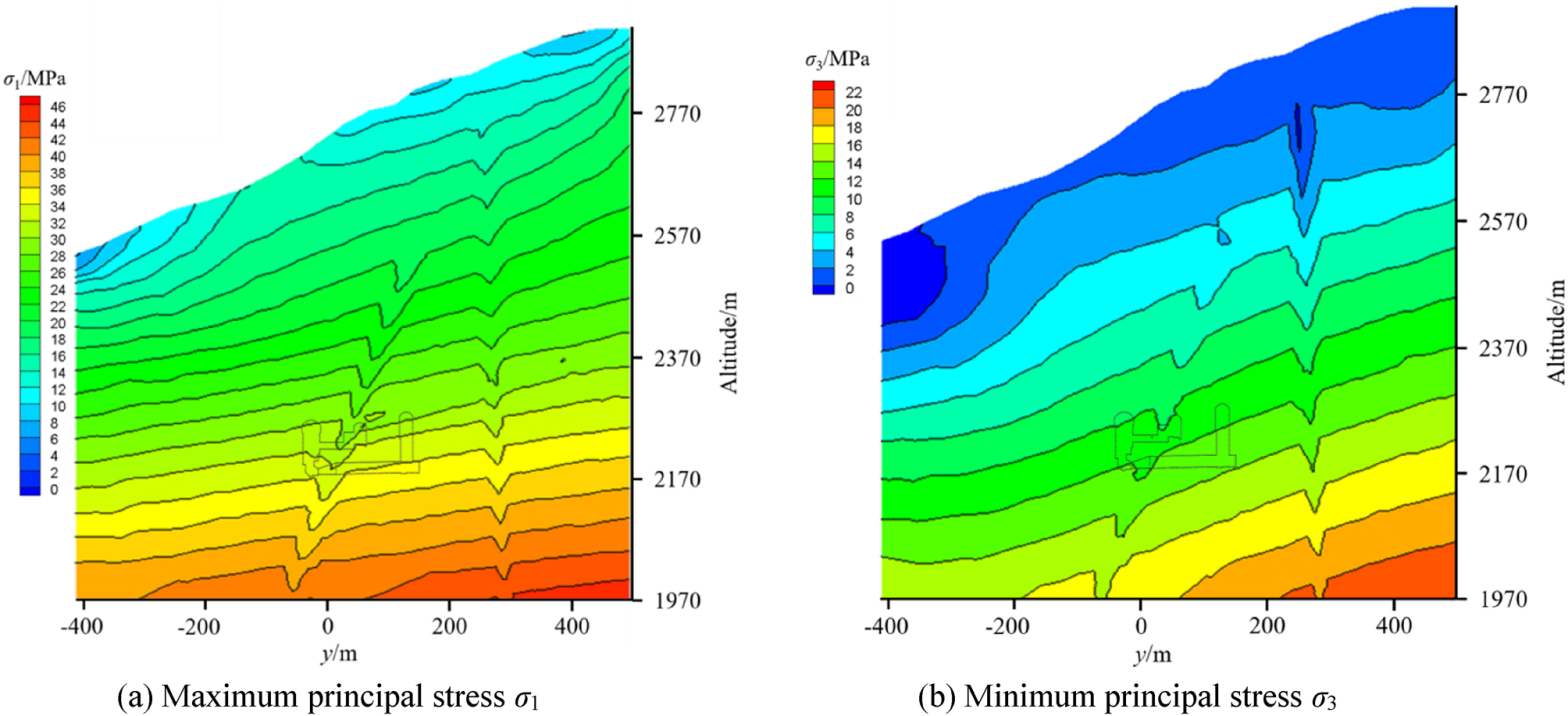
Principal stress contour at *x* = 0.

Based on the calculated present stress field, the contour maps of *y*′ = 0 in Fig. 3 including some measured points are shown in Fig. 9. The topography has a significant influence on the in situ stress distribution in the engineering area, while the lithology has a small influence on the stress field. From the top to the bottom of the Earth’s surface, *σ*_*x*_, *σ*_*y*_ and *σ*_*z*_ increase with increasing depth. Due to the weak mechanical parameters at faults and lamprophyre, there are stress release effects in these areas. The stress components of the faults and lamprophyre all drop sharply, and the stress components of nearby rock masses also decrease. Figure 10 shows the principal stress contour map of Unit 1 in the main powerhouse, indicating that.

1. The principal stress changes with the topography, and the surface unloading area has a small deformation modulus due to the relaxation of the rock mass structure, so there is a stress relaxation.
2. When the horizontal and vertical burial depths increase, the values of *σ*_1_ and *σ*_3_ gradually increase, and the contour lines are roughly parallel to the hillside.
3. With the increase in the depth of the caves, the stress value of the cave surrounding rock also increases, and the stress of the tailwater surge chamber is higher than that of the main powerhouse. The excavation and unloading stresses at the lower part of each cave are higher than those at the top arch, indicating that the excavation unloading effect of the lower part of the cave is more pronounced.
4. The stress field presents distribution characteristics of *σ*_*x*_ > *σ*_*y*_ > *σ*_*z*_, which is advantageous to excavation of the cave sidewalls, but the stability of the end wall is unfavorable.

**Figure 11:**
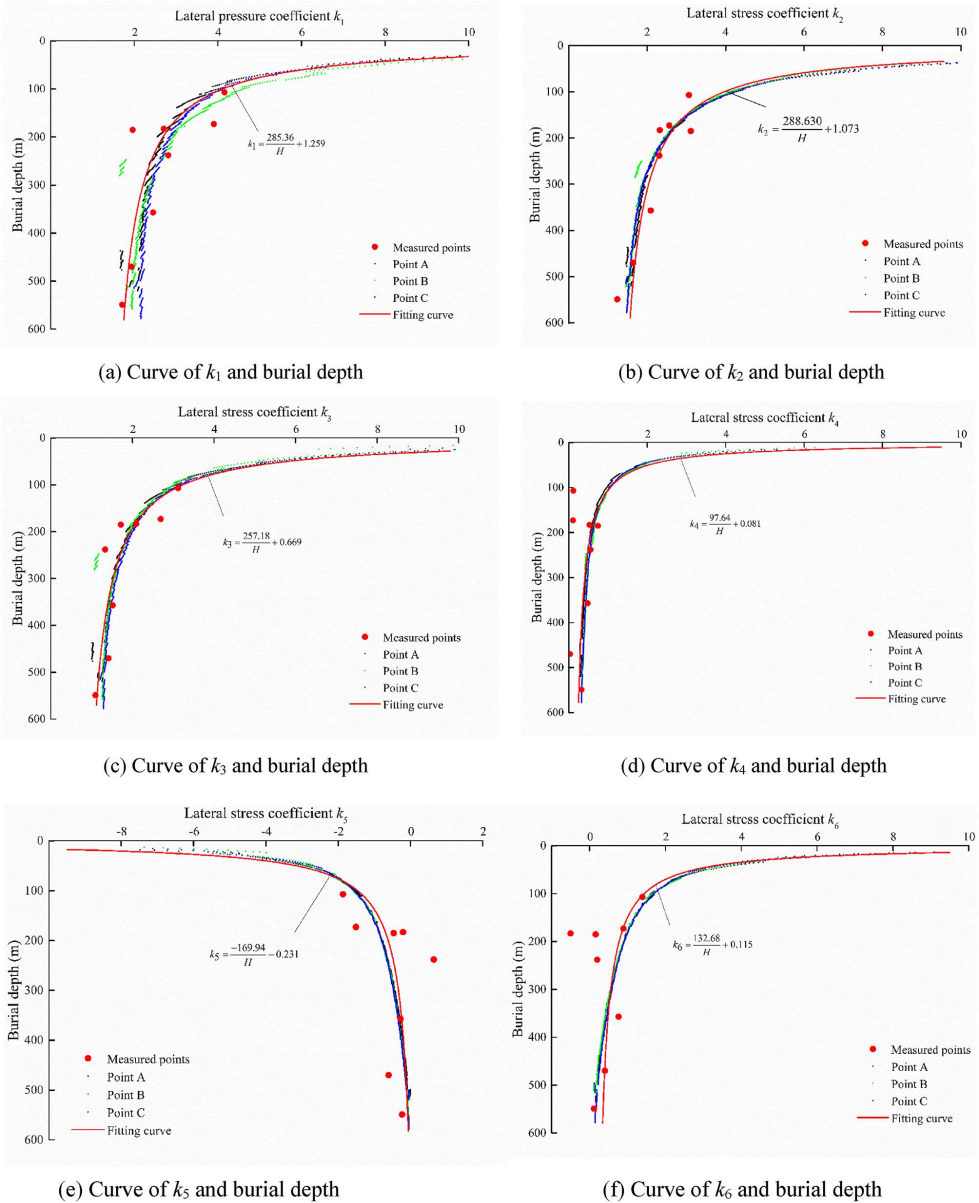
Relationship between *k* and burial depth.

The lateral stress coefficients *k*_1_*–k*_6_ along the perpendicular line through Points A, B and C in Fig. 5 of the center of the three caverns are shown in Fig. 11. The calculated values of the lateral stress coefficients are close to the measured points but disperse from the main curve when the location is at the fault or lamprophyre. The lateral stress coefficients decrease with increasing burial depth and gradually tend to a constant value. The fitting curve shows the relationship between burial depth and lateral pressure coefficient Points A, B and C. The relationship is in accordance with Eq. (3), which can be directly imported into the numerical model with a cavern group for excavation simulation.

## Conclusion

The in situ stress field of the Shuangjiangkou Hydropower Station is mainly composed of tectonic stress and gravity stress, and its distribution is affected by the tectonic stress, geological structure and topography. In this paper, the ancient stratum excavation method combined with the lateral stress coefficient and GAN is utilized to invert the in situ stress field. By comparing with measured points, the feasibility of this method to invert the in situ stress field is verified.

1. The in situ stress value is positively correlated with the burial depth and the lateral stress coefficient. For deep buried engineering, the lateral stress coefficient is inversely proportional to the burial depth and tends to a constant value, rather than following the commonly used linear relationship.
2. Instead of inverting the stress at measured points with the artificial intelligence method, the regression factor of the lateral stress coefficient in ancient times is determined by GAN. Then, the in situ stress field is obtained by strata excavation to simulate the geological effects of surface erosion and weathering to inverse the present in situ stress field.
3. Elements containing both rock masses and faults are regarded as equivalent elements of transverse isotropy, thereby reducing the difficulty of numerical model modeling and calculation time.
4. The inverted in situ stress components are close to the measured values, so the obtained in situ stress field is reasonable; thus, the inverted in situ stress field can reflect the distribution law of the actual in situ stress field and can provide reasonable guidance for the excavation simulation and stability analysis of underground caverns.
5. Based on the in situ stress field obtained by the inversion analysis, the equation of the lateral stress coefficient and the burial depth can be obtained. In subsequent cavern excavation simulations, a refined model with a cavern group can be established, and the reversed geostress equation can be directly imported into the model.

In summary, this paper proposes a new inversion method of the in situ stress field, which can provide a more optimized in situ stress field for deep-buried engineering.
